# Knowledge, Attitude and Perception of Ebola Virus Disease among Secondary School Students in Ondo State, Nigeria, October, 2014

**DOI:** 10.1371/currents.outbreaks.c04b88cd5cd03cccb99e125657eecd76

**Published:** 2016-03-04

**Authors:** Olayinka Ilesanmi, Faith Osaretin Alele

**Affiliations:** Department of Community Health, Federal Medical Centre, Owo, Ondo State, Nigeria; Department of Community Health, Federal Medical Centre, Owo, Ondo State, Nigeria

**Keywords:** ebola, Ebola Virus Disease, infection, student

## Abstract

Introduction: The first case of Ebola Virus Disease (EVD) in Nigeria was imported on 20th July 2014, by an air traveller. On 8th August, 2014, WHO declared the Ebola outbreak in West Africa a Public Health Emergency of International Concern (PHEIC). This study aimed at assessing the knowledge, perception and attitude of secondary school students towards EVD and adopting disease preventive behaviour.

Methods: A descriptive cross sectional study of 440 students from a mixed secondary school in Owo, Ondo State was done. Data was collected in October 2014 when Nigeria was yet to be declared EVD free.Simple random sampling was used to select the school while Systematic random sampling was used in the selection of participants. A semi-structured, interviewer administered questionnaire was used to collect data. Data was analyzed with SPSS version 21. Descriptive statistics and Chi-square test were done, level of statistical significant was 5%.

Results: Mean age of respondents was 13.7±1.9 years. Females were 48.2%. Most of the respondents had heard of Ebola Virus Disease (95.4%). Female respondents (51.3%), those who were 15 years and above (51.1%) and in the senior class (54.1%), and had good general knowledge of EVD and across all domains. Being in the senior secondary class and seeking for health care in the hospital were positively associated with good general knowledge (p-value: 0.029, and <0.001 respectively). Three commonest modes of spread of EVD mentioned were contact between infected animals and men (74.8%), touching body fluids of a person who is sick of EVD (57.0%), and contact (55.2%). The top three signs of EVD mentioned were abnormal bleeding from any part of the body (56.10%), vomiting (47.0%) and fever (42.3%).

Conclusion: Our results revealed suboptimal EVD-related knowledge, attitude and practice among the students. Promotion of health messages and training of students on prevention of EVD to effectively control past and future outbreaks of EVD in Nigeria was immediately initiated in schools in Ondo State.

## Introduction

Ebola virus disease (EVD), previously known as Ebola hemorrhagic fever is a rare and deadly acute viral illness caused by Filoviridae family. Ebola can cause disease in both humans and nonhuman primates (monkeys, gorillas, and chimpanzees). EVD causes severe and often fatal illness in humans and has a case fatality rate of up to 90% if left untreated^1^^,^ 2. The first outbreak of the disease was reported in 1976, in two simultaneous outbreaks in Yambuku, Democratic Republic of Congo in a village situated near the Ebola River, from which the disease derives its name and in Nzara, Sudan^2^^,^ 3. The natural reservoir of Ebola virus is unknown and it is spread through direct contact with blood or body fluids of a person who is sick with Ebola, objects (like needles and syringes) that have been contaminated with the virus, and infected fruit bats or primates (apes and monkeys) 1. The disease is often characterised by a range of non-specific symptoms such as muscle pain, headache, sudden onset of fever, intense weakness, sore throat, followed by vomiting, diarrhoea, and impaired kidney and liver function with resultant end organ damage 2. About 30%–50% of EVD patients experience haemorrhagic symptoms and till the end of 2014 there was no approved antiviral drug or vaccine for the treatment and prevention of EVD 4.

The 2014-2015 outbreak in West Africa, first reported in Guinea in March 2014 was the largest and most complex Ebola outbreak in history with a combined total of 528 cases (including laboratory-confirmed, probable, and suspected cases) and 337 deaths (case-fatality rate = 64%) reported in three countries (Guinea, Liberia and Sierra Leone) as of 18th of June, 2014 5. As at the 22nd September, 2015, a total of 28295 cases with 11295 deaths were recorded in the three West African Countries 6. The disease is a great source of public health concern and poses as an immigration risk to unaffected countries. Factors such as lack of knowledge, limited or lack of infection prevention and control resources, poor public health infrastructure and highly transmissible nature of the virus have been shown to increase the outbreak of the disease 7.

The first Ebola virus disease case in Nigeria was recorded on the 20th of July 2014 with the arrival of an acutely ill air traveller from Liberia at the international airport in Lagos. Prior to this Nigeria had never had any case of EVD and this created a lot of fear and panic across every fabric of the society especially with the paucity of information about the disease and no known proven treatment for the disease. This index case resulted in an outbreak with 19 EVD cases and 8 deaths reported 8. Nigeria was declared EVD free by WHO on 20th October 2014 after no new cases had been detected for 42 days 9. This was as a result of the collaborative efforts of the Federal Ministry of Health /Nigerian Centre for Disease control (FMOH/NCDC), the Lagos State Ministry of Health and partner agencies which established an Ebola Emergency Operations Centre (EEOC) to coordinate all outbreak response activities 8. Other measures instituted were the use of available social and mass media to spread information about EVD and to promote preventive measures such as personal hygiene, and good public health practices 10. A toll-free Ebola help phone number was established and made available to the general public to seek medical advice and care when the need arises.

The study aimed at assessing the knowledge, perception and attitude of students towards EVD and adoption of disease preventive behaviour.

## Methods

The study area was one of the secondary schools in Owo. All consenting selected students were studied. A descriptive cross sectional design was used.

**Sampling methods**

Stage 1: From the list of all public secondary schools in Owo metropolis, number were assigned to each of the schools, one was selected by simple random sampling. The school corresponding to the selected number was chosen and the study was carried out in the selected school.

Stage 2: From the list of students in each class systematic sampling was used to select participants until the required sample size was achieved.

**Sample size determination**

The sample size was calculated using the Leslie Kish formula for sample size determination for proportion. Minimum desired sample size calculated was 402 with a prevalence of 50%, using the standard normal deviate of 1.96 which corresponds to 5% level of significance.

**Data management**

A semi-structured interviewer administered questionnaire was used. Questionnaires were checked for omissions and errors after collection and correction were made where necessary. Data was analysed with SPSS version 21, descriptive statistics were done, Chi square test was used for the assessment of significant associations between proportions at 5 % level of significance. Knowledge was graded based on three EVD domains; mode of spread, symptoms and signs, and preventive measures. Scores were assigned to correct responses mentioned by the respondents. Respondents who scored 4 points and above in each EVD domain were considered to have had good knowledge of the domain. Respondents with a total score of 12 and above in the three domains were categorized as having overall good general knowledge.

**Ethical consideration**

Ethical approval for the study was sought from Health Research Ethics Committee, Federal Medical Centre, Owo, Ondo State. Participants were made to understand that participation is voluntary and there were no consequences for non-participation. All information obtained was kept confidential.

## Results

The mean age of respondent was 13.7 years ± 1.9 standard deviation. Age ranged from 10-19 years. Only 133(30.23%) were 15 years and above. Females were 212 (48.18%) Other sociodemographic characteristics are shown in table 1.

**Table 1: Socio-demographic characteristics of secondary school students in Ondo State:** 

Socio-demographic Characteristics	Frequency	Percent
Age group in years		
<15	307	69.77
15 and above	133	30.23
Sex		
Female	212	48.18
Male	228	51.82
Ethnicity		
Yoruba	363	82.50
Others	77	17.50
Religion		
Christianity	367	83.41
Islam	73	16.59
Class		
Junior	220	50.00
Senior	220	50.00

Most of the respondents said they had heard of Ebola Virus Disease (95.4%). Respondents who were 15 years and above (51.1%), in the senior class (54.1%) had good general knowledge of EVD and across all domains, while female respondents had better general/overall knowledge of EVD (51.3%) and in all the domains compared with their male counterparts. Table 2 shows the association between socio-demographic characteristics and knowledge score of respondents on EVD in all domains,

**Table 2: Association between socio-demographic characteristics and knowledge score of respondents on EVD across all domains:** 

Respondents with good knowledge across all domains				
Socio-demographic Characteristics	Mode of spread n(%)	Symptoms and signs n(%)	Preventive measures n(%)	General/overall knowledge n(%)
Age group in years				
<15	121(41.9)	121(43.4)	138(47.8)	133(47.7)
15 and above	62 (47.3)	63 (48.1)	66 (50.4)	67 (51.1)
Sex				
Female	93(46.7)	94 (48.2)	104(52.3)	100(51.3)
Male	90 (40.7)	90 (41.9)	100(46.5)	100(46.5)
Ethnicity				
Yoruba	147(42.1)	149(44.0)	164(47.0)	157(46.3)
Others	36 (50.7)	35 (49.3)	40 (56.3)	43(60.6)
Religion				
Christianity	161(45.4)	166(47.7)	30 (46.2)	176(50.6)
Islam	22 (33.8)	18 (29.0)	174(49.0)	24(38.7)
Class				
Junior	80 (38.3)	78 (38.4)	93 (44.5)	88(43.3)
Senior	103(48.8)	106(51.2)	111(52.6)	112(54.1)

The three commonest modes of spread of EVD mentioned by the respondents were contact between infected animals and men (74.8%), touching body fluids of a person who is sick of EVD (57.0%), and contact with a person who is sick of EVD (55.2%). 33.2% mentioned participation in the burial rites of a person who died of EVD as possible modes of spread of the disease while approximately 32% of respondents mentioned contact with clothing, beddings and other utensils of a person who is sick of EVD.

The top three signs of EVD mentioned by the respondents were abnormal bleeding from any part of the body (56.10%), vomiting (47.0%) and fever (42.3%) as seen in figure 1.

**Signs and symptoms of EVD mentioned by respondents:**
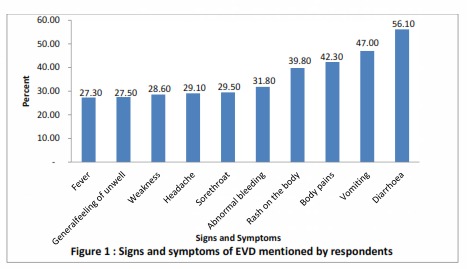

Table 3 shows the association between the level of knowledge on EVD and key characteristics, respondents in the senior class and those who agreed to seek for care in the hospital were more likely to possess good knowledge of EVD ( p-value: 0.029 and <0.001 respectively).

**Table 3: Association between level of knowledge on EVD and key characteristics:** 

Socio-demographic Characteristics	Good knowledge n(%)	Poor knowledge n(%)	Chi Square	p value
Age group in years				
<15	133 (47.7)	146 (52.3)	0.431	0.512
15 and above	67 (51.1)	64 (48.9)		
Sex				
Female	100 (51.3)	95(48.7)	0.931	0.335
Male	100 (46.5)	115(53)		
Ethnicity				
Yoruba	157 (46.3)	182 (53.7)	0.677	0.410
Others	43 (60.6)	28 (39.4)		
Religion				
Christianity	176 (50.6)	172 (49.4)	2.965	0.085
Islam	24 (38.7)	38 (61.3)		
Class				
Junior	88 (43.3)	115 (56.7)	4.746	0.029
Senior	112 (54.1)	95 (45.9)		
Hand-washing practice				
Poor	0 (0.0)	4 (100.0)	Fischer’s	0.057
Good	214 (51.4)	202 (48.6)	Exact test	
EVD care-seeking behaviour				
Will not go to the hospital	27 (29.7)	64 (70.3)	21.867	<0.001
Will go to the hospital	180 (57.5)	133 (42.5)		

## Discussion

The public health significance of EVD lies in its rarity, lack of effective therapeutic and prophylactic measures and its potential to cause significant morbidity and mortality especially during outbreaks 11. The fear of EVD during the last outbreak in Nigeria affected and threatened the social fabric of the nation. There were behavioural modifications as regards the health of individuals and the community at large which unfortunately has not been sustained after Nigeria was declared Ebola virus disease free. The social media was agog with messages about self-protective measures such as regular hand washing, use of hand sanitizers and reporting any form of fever or illness to the nearest hospital 10. There have been no previous occurrence of EVD in Nigeria, therefore nothing has been documented about the knowledge of students and their attitudes towards the disease. Literature review on EVD also revealed limited data published in Nigeria.

Most of the respondents in the studied population reported that they had heard of EVD which was expected with the jingles about the disease on the social and mass media. A KAP study conducted in three different states in Nigeria also revealed that majority of the respondents reported that they had also heard of EVD 13. Respondents who were females and older in age had good general knowledge of EVD and were more likely to adopt appropriate measures to prevent community spread 14. More respondents reported that contact with an infected animal as a mode of spread while only a few mentioned contact with fomites and participation in burial of a person who died of EVD. Similar result was obtained among respondents in Kano State of Nigeria where only a few were aware of the risk of transmission during funeral rites 13. The latter mode of spread is more important because of the risk of transmission associated with funeral rites 15. These findings proved that modification of communication messages that focused on detailed education of the public about EVD, its mode of spread, and prevention should have been done as part of the on-going measure to contain the outbreak.

In this study, it was found out that respondents who were in the senior secondary class were found to have significantly good knowledge of EVD compared to those in the junior secondary class. An association has been established between education and health; the higher the level of education, the better the health seeking behaviour and healthier outcomes 16. Also, those who agreed to visit the hospital to seek care were found to have significantly good knowledge of EVD compared to their counterparts. A high index of suspicion is needed to promptly identify EVD cases to reduce the risk of community spread of the disease; public health education should be intensified while clearly spelling out the modes of transmission, and preventive measures.

## Conclusion

We recommended the promotion and sustainability of health messages focusing on the mode of transmission and preventive measures such as demonstration of hand-washing techniques, addressing myths and misconceptions; and promoting safe burial practices. The public health action that followed this survey was the commencement of school health education and training of students on prevention of EVD in Owo Local Government Area of Ondo State, Nigeria. This should be extended to other schools across Nigeria.

## Competing Interests

The authors have declared that no competing interests exist.
